# An Acute Transverse Myelitis Attack after Total Body Irradiation: A Rare Case

**DOI:** 10.1155/2013/523901

**Published:** 2013-07-24

**Authors:** Muzaffer Keklik, Leylagul Kaynar, Afra Yildirim, Serdar Sivgin, Celalettin Eroglu, Serife Cingoz, Cigdem Pala, Bulent Eser, Mustafa Cetin, Ali Unal

**Affiliations:** ^1^Erciyes Stem Cell Transplantation Hospital, Department of Hematology, Faculty of Medicine, Erciyes University, 38039 Kayseri, Turkey; ^2^Department of Radiology, Faculty of Medicine, Erciyes University, 38039 Kayseri, Turkey; ^3^Department of Radiation Oncology, Faculty of Medicine, Erciyes University, 38039 Kayseri, Turkey; ^4^Department of Internal Medicine, Faculty of Medicine, Erciyes University, 38039 Kayseri, Turkey

## Abstract

Total body irradiation (TBI) combined with chemotherapy is widely used as a pretreatment regimen of bone marrow transplantation (BMT) in hematologic disorders. Late complications related to TBI as part of the conditioning regimen for hematopoietic stem cell transplantation have been revealed. Acute transverse myelitis (ATM) is a neurological syndrome characterized by disorder of motor, sensorial, and autonomic nerves, and tracts at medulla spinalis, which is resulted from involvement of spinal cord. In this paper, we presented an ATM attack developed after TBI in a patient with acute lymphoblastic leukemia (ALL) as it is a rarely seen case.

## 1. Introduction 

With the increasing use of bone marrow transplantation (BMT) for hematologic malignancies, total body irradiation (TBI) is becoming a more commonplace procedure. The goal from using TBI in the preparation regimens for BMT is threefold: destroying residual neoplastic cells, clearing the host marrow to allow repopulation with donor marrow cells, and providing sufficient immunosuppression to avoid allograft rejection by immunologically active cells in the host [1]. Known adverse effects of TBI include nausea, vomiting, sweating and irritability as well as renal dysfunction, interstitial pneumonia, lung injury, cataract, osteosarcoma, and cystitis in rare cases [2–8]; no case of acute transverse myelitis (ATM) has been reported. ATM is a neurological syndrome caused by inflammation of spinal cord, which may involve all age groups. Estimated annual number of cases with ATM varies from 1 to 5 per million cases. In the present study, we presented an ATM attack developed after total body irradiation (TBI) in an acute lymphoblastic leukemia (ALL) case, as it has not been found in the literature so far. 

## 2. Case

**Figure 1:**
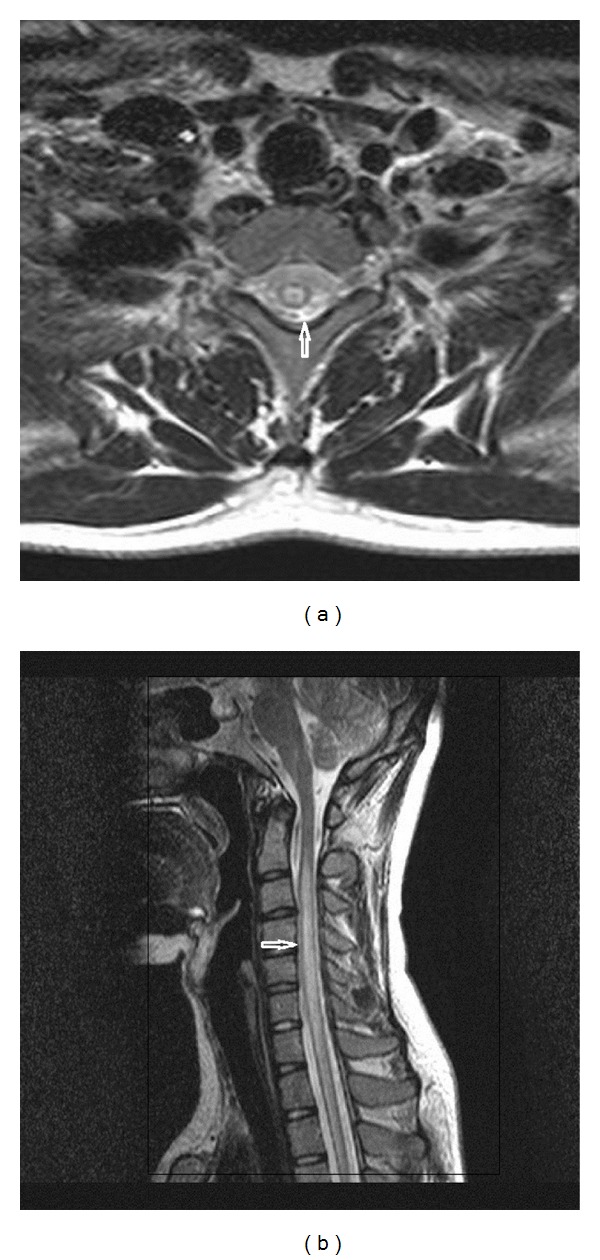
T2-weighted axial (a) and sagittal (b) MR images of the cervical spine demonstrate long segmental intramedullary high signal intensity suggesting transverse myelitis.

**Figure 2:**
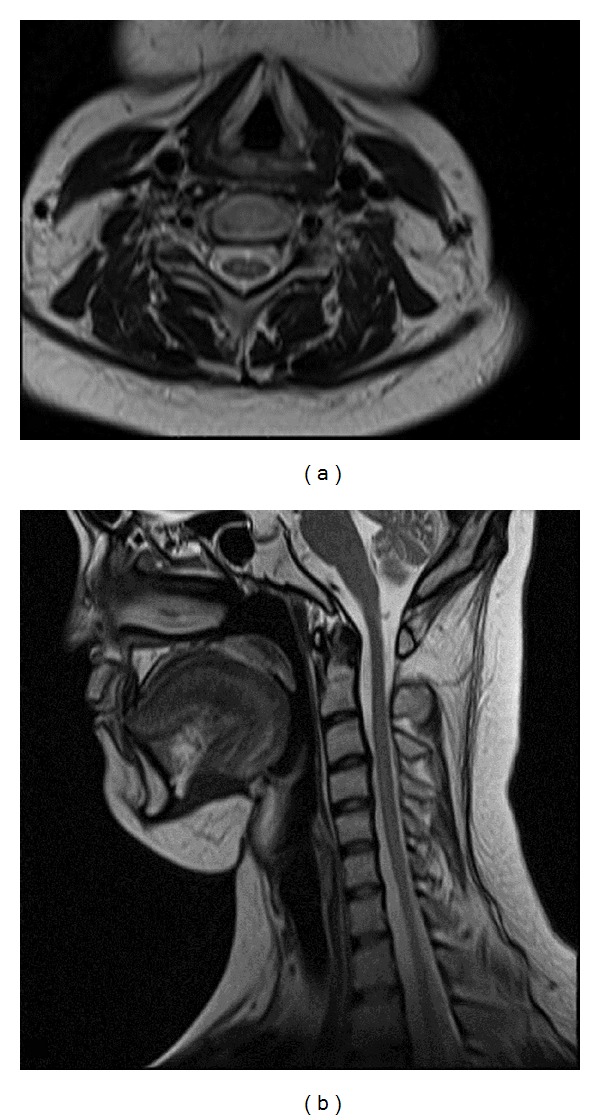
Posttreatment T2-weighted axial (a) and sagittal (b) MR images show normal signal intensity of cervical spinal cord.

 An 18-year-old man was presented with fatigue, loss of appetite, and abdominal distension at 2004, when he was 10 years old. In the physical examination, splenomegaly was detected and the following findings were recorded in blood tests: hemoglobin (Hb), 8.5 g/dL, white blood cells (WBC), 69.9 × 10^9^/L, and platelet count (Plt), 354 × 10^9^/L. Philadelphia (Ph) (+) chronic, stable-phase chronic myeloid leukemia (CML) diagnosis was made by peripheral blood smear and bone marrow evaluations including morphological and genetic studies. After cytoreduction by hydroxyurea, he was given 400 mg/day imatinib therapy; however, there was loss of hematological response for three times during 2004–2009 period; thus, his treatment was converted to dasatinib therapy at 2009. At 2010, dasatinib 1 × 70 mg plus induction chemotherapy protocol was given to Ph (+) patient with common ALL antigen (CALLA) (+) pre-B ALL: methotrexate 15 mg per day intrathecally on day 1, vincristine 2 mg per day intravenously on days 4, 11, and 18, dexamethasone 10 mg/m^2^ per day intravenously on days 4, 5, 11, 12, 13, and 14, daunorubicin 45 mg/m^2^ per day via intravenous infusion on days 4, 5, 11, and 12, and L-asparaginase 500 u/m^2^ per day via intravenous infusion over 2 h on day 18. As the consolidation therapy, the patient received methotrexate 1350 mg/m^2^ per day via intravenous infusion over 24 hours on day 1, cytarabine 2000 mg/m^2^ per day via intravenous infusion over 12 hours on day 5, vincristine 2 mg per day via intravenous route on day 1, dexamethasone 10 mg/m^2^ per day via intravenous route on days 1–5, etoposide 250 mg/m^2^ per day via intravenous infusion over 1 hour on days 4-5, and methotrexate 15 mg per day via intrathecal route on day 1. Allogeneic hematopoietic cell transplantation (allo-HCT) decision was taken for patient who had bone marrow remission following remission induction and consolidation therapies and 10/10 fully compatible unrelated donor. According to allo-HCT preparation protocol, TBI (2 Gy, twice daily for 3 days; overall dose 12 Gy) followed by 60 mg/kg/day over 2 days cyclophosphamide regime were planned; however, he reported that he could not move his arms on the day 3 of TBI therapy. He had no symptom other than neurological complaint. There was no abnormal finding in physical examination, but 3/5 weakness was detected in both upper extremities in neurologic examination, as being more prominent in distal. No pathological reflex was detected. Deep tendon reflexes were hypoactive at upper extremities, but there was no sensorial defect. Thus, TBI was discontinued and 1 mg/kg prednisolone intravenous treatment was initiated. Cerebrospinal fluid evaluation was negative for malignancy or infectious pathogens. On the cervical magnetic resonance imaging (MRI), there were T1-weighted isointense and T2-weighted hyperintense pathological signal changes, which begin at C2 level, involving all cervical spinal cord and extends to thoracic segments and expansion of spinal cord (Figure 1). By these findings, diagnosis of ATM was made. No abnormal finding was detected in viral, bacteriological, and autoimmune evaluations; in addition, no growth was found in culture tests: cytomegalovirus (CMV), rubella virus, herpes simplex virus (HSV), varicella zoster virus (VZV), and Epstein-Barr virus evaluations; hepatitis markers and brucellosis test were found negative. Antinuclear antibody (ANA), antidouble-stranded DNA (anti-dsDNA), antismooth muscle antibody (Anti-Sm), antitopoisomerase I (anti-Scl-70) antibody, anti-Sjögren's syndrome A (anti-SSA), and anti-Sjögren's syndrome B (anti-SSB) were also negative. Serum angiotensin converting enzyme (ACE), C-reactive protein (CRP), and erythrocyte sedimentation rate (ESR) values were in normal range. Symptoms of the patient were completely resolved 36 hours after cessation of TBI. On the posttreatment cervical MRI, there was normal signal intensity of cervical spinal cord (Figure 2). Steroid therapy was also discontinued after resolution of the symptoms. 

No complication other than neutropenic fever was observed during transplantation period. On day 35, graft-versus-host-disease (GVHD) was developed involving skin (Grade I) and liver (Grade I). Thus, cyclosporine and steroid therapy was initiated. On day 91, chimerism result was found as 100% in the patient on dasatinib therapy (1 × 100 mg). The patient is being followed with hematological and molecular remission (Philadelphia PCR: negative). 

## 3. Discussion

TBI is a complex technique used for treating hematologic diseases. The role of TBI is to destroy the recipient's bone marrow and tumor cells and to immunosuppress the patient sufficiently to avoid rejection of the donor's bone marrow transplant [9]. Radiation myelitis (RM) is a clinical presentation which can develop as a result of temporary demyelination caused by the radiation-related inhibition of oligodendroglial cells that produce myelin within spinal cord segment irradiated, and it is defined as early or late phase radiation myelitis [10]. The factors involved in the development of RM include length of spinal cord irradiated and radiation dose used. TBI-related RM is a rare entity, which is reported in patients received radiotherapy that excess tolerable doses at involved field [11]. In our patient, there was no history of previous radiotherapy. ATM cases are most commonly seen at 10–19 and 30–39 years of age; it has no familial tendency or gender predominance. Our case was 18-year-old man without family history in terms of ATM. Etiological factors include bacterial and viral infections, vaccines, systemic autoimmune diseases (systemic lupus erythematosus, Sjögren's syndrome, sarcoidosis, and multiple sclerosis), paraneoplastic syndromes, and spinal vascular events; however, there is considerable number of idiopathic cases [11]. We evaluated etiological factors in our case. Regarding infection, no fever was observed, while CRP and ESR were in normal range. The patient received no vaccination during hospitalization period. All viral, bacterial, and autoimmune evaluations were negative. No growth was detected in culture tests. Thus, we considered that the case might be an ATM attack caused by TBI. Schwartz et al. revealed that, as RM is not associated with any pathognomonic features, it can be entertained only after all infectious, metastatic, neurologic, and orthopedic etiologies have been excluded [12]. They suggested that opportunistic leptomeningitis (VZV, CMV, aspergillosis, tuberculosis, and bacterial), epidural or vertebral metastasis, congenital or acquired spine malformations, preexisting central nervous system damage, and intracranial/spinal infarction secondary to an autoimmune, vasculitic, or hypercoagulable state must all be considered. In our patient, there was no clinical evidence to suggest any of these causes. ATM attack attributed TBI was dramatically resolved 36 hours after cessation of TBI. In the ATM, there is an involvement of motor and sensorial tractus across a certain segment in medulla spinalis, and clinical findings may vary depending on the area involved. Symptoms depend on the level of spinal cord involved, including weakness or sensorial deficit at upper and lower extremities, dysfunction of intestine or bladder, back pain, and radicular pain. Up to 45% of patients may worsen at the maximum level. Most patients have weakness at lower limb with varying degrees. The weakness of upper extremity seen in our case is encountered in the limited number of the cases and depends on the level of medulla spinalis involvement. Treatment depends on etiology; however, steroid therapy is recommended in idiopathic cases. The resolution may never occur; it may be either partial or complete; and it usually begins at months 1 and 3. In our case, there was no neurological finding other than weakness of upper extremity. In addition, no fecal or urinary incontinence developed. Moreover, the findings were resolved 36 hours after cessation of TBI. We think that steroid therapy rapidly controlled the neurological findings. Rapid resolution of symptoms by steroid therapy supports the accuracy of the diagnosis. In fact, steroid therapy is recommended in the treatment of ATM. In conclusion, we think that our case is a rare ATM attack developed after TBI.
